# Molecular Size Modulates Pharmacokinetics, Biodistribution, and Renal Deposition of the Drug Delivery Biopolymer Elastin-like Polypeptide

**DOI:** 10.1038/s41598-018-24897-9

**Published:** 2018-05-21

**Authors:** Marija Kuna, Fakhri Mahdi, Alejandro R. Chade, Gene L. Bidwell

**Affiliations:** 1 Department of Cell and Molecular Biology, University of Mississippi Medical Center, Jackson, MS USA; 2 Department of Neurology, University of Mississippi Medical Center, Jackson, MS USA; 3 Department of Physiology and Biophysics, University of Mississippi Medical Center, Jackson, MS USA; 4 Department of Medicine, University of Mississippi Medical Center, Jackson, MS USA; 5 Department of Radiology, University of Mississippi Medical Center, Jackson, MS USA

## Abstract

Elastin-like polypeptides (ELP) are engineered proteins that consist of repetitions of a five amino acid motif, and their composition is easily modified to adjust their physical properties and attach therapeutics. Because of the repetitive nature of the ELP sequence, polymer size is particularly amenable to manipulation. ELP fusion proteins are being actively developed as therapeutics for many disease applications, and how the ELP size and shape affects its pharmacokinetics and biodistribution is a critical question for the general field of ELP drug delivery. To address this, we generated a library of ELPs ranging in size from 25 kDa to 110 kDa. Terminal plasma half-life was directly proportional to polymer size, and organ biodistribution was also size dependent. The kidneys accumulated the highest levels of ELP of all sizes, followed by the liver. Within the kidney, most ELP was found in the proximal tubule, but intra-renal localization shifted from exclusively cortical to a mixture of cortical and medullary as ELP size increased.

## Introduction

Elastin-like polypeptides (ELPs) are genetically engineered proteins utilized as a delivery system for therapeutics^1–12^. They were initially characterized by Urry and colleagues^13,14^, who described their biophysical properties. ELPs consists of five amino acid repeats (pentamers), Val–Pro–Gly–Xaa–Gly, where Xaa is a guest residue and can be any amino acid except proline. The amino acid sequence, together with the number of pentamer repeats, defines the ELP molecular weight and transition temperature (T_t_)^15^. T_t_ is the temperature at which ELPs undergo a phase transition during which soluble ELP molecules self-associate to form aggregates. The aggregation process is fully reversible, and this unique physical property has been exploited to facilitate ELP purification^16,17^ and for drug delivery purposes^12^. Modifications of the sequence composition and length can be achieved by recursive directional ligation^18^, and their influence on the polypeptide’s T_t_ have been extensively studied^15,18,19^.

Furthermore, the ELP sequence is easily modified to include therapeutic peptides and proteins, and small molecule drugs can easily be chemically attached. These ELP fusions confer increased stability to therapeutic peptide and protein cargo, and they can increase solubility and reduce off-target toxicity of small molecule drugs. ELPs can be engineered to form nanoparticles for specialized drug delivery applications or to form hydrogels for controlled drug release^20–24^. Their thermal responsiveness can be exploited for targeted drug delivery using externally applied focused hyperthermia^25^.

The versatility of ELPs has led to their development as drug carriers in many different disease areas. However, careful analysis of how their physical properties, including chain length and hydrodynamic radius, influence their *in vivo* behavior has not been systematically described. A study by Dreher *et al*. investigated how molecular weight influences accumulation of a model macromolecular drug carrier, dextran, in tumors^26^. They found that an increase in the dextran MW significantly decreased their vascular permeability, yet it increased their plasma half-life, and that the highest tumor accumulation was achieved with dextran between 40–70 kDa. Also, using three different sizes of ELP in a mouse cancer model, Ryu *et al*. showed that total tumor accumulation is dependent on ELP molecular weight^27^. Much focus on ELP has been for development of targeted tumor drug delivery, but ELPs are being used in a myriad of applications outside of the cancer field^12^. How ELP size and shape affects pharmacokinetics and biodistribution in non-cancer models is a critical question for the general field of ELP drug delivery.

Our lab has used ELP-fusion proteins extensively for renal drug delivery. We previously created an ELP fusion with vascular endothelial growth factor (VEGF) for therapeutic angiogenesis to treat renovascular disease. We showed that the ELP-VEGF chimera maintains full potency *in vitro* and has improved pharmacokinetic parameters *in vivo*^17^. Furthermore, intrarenal or systemic intravenous administration of ELP-VEGF in a swine model of renovascular disease improved renal blood flow and glomerular filtration rate with a corresponding increase in renal microvascular density^9,28^.

In this study, we aimed to define the effects of molecular weight on the pharmacokinetics, biodistribution, and renal deposition of ELP and to determine the molecular weight best suited for renal drug delivery. We hypothesize that with an increase in MW, ELPs will have an increased half-life and tissue accumulation; smaller ELPs will be rapidly filtered by the kidneys, while larger ELPs won’t be able to cross the filtration barrier and will remain in blood longer, providing more time to accumulate in other tissues. Furthermore, this work could lead to identification of ELP constructs that target only pre-glomerular structures or that simultaneously target both pre- and post-glomerular renal targets. Beyond our specific application to renal drug delivery, this study also provides a detailed characterization of how ELP chain length affects the protein’s pharmacokinetics and biodistribution, which is critical information when developing ELPs as drug carriers for many different disease applications.

## Results

### Synthesis of an ELP Library with Varying Molecular Weights

**Figure 1:**
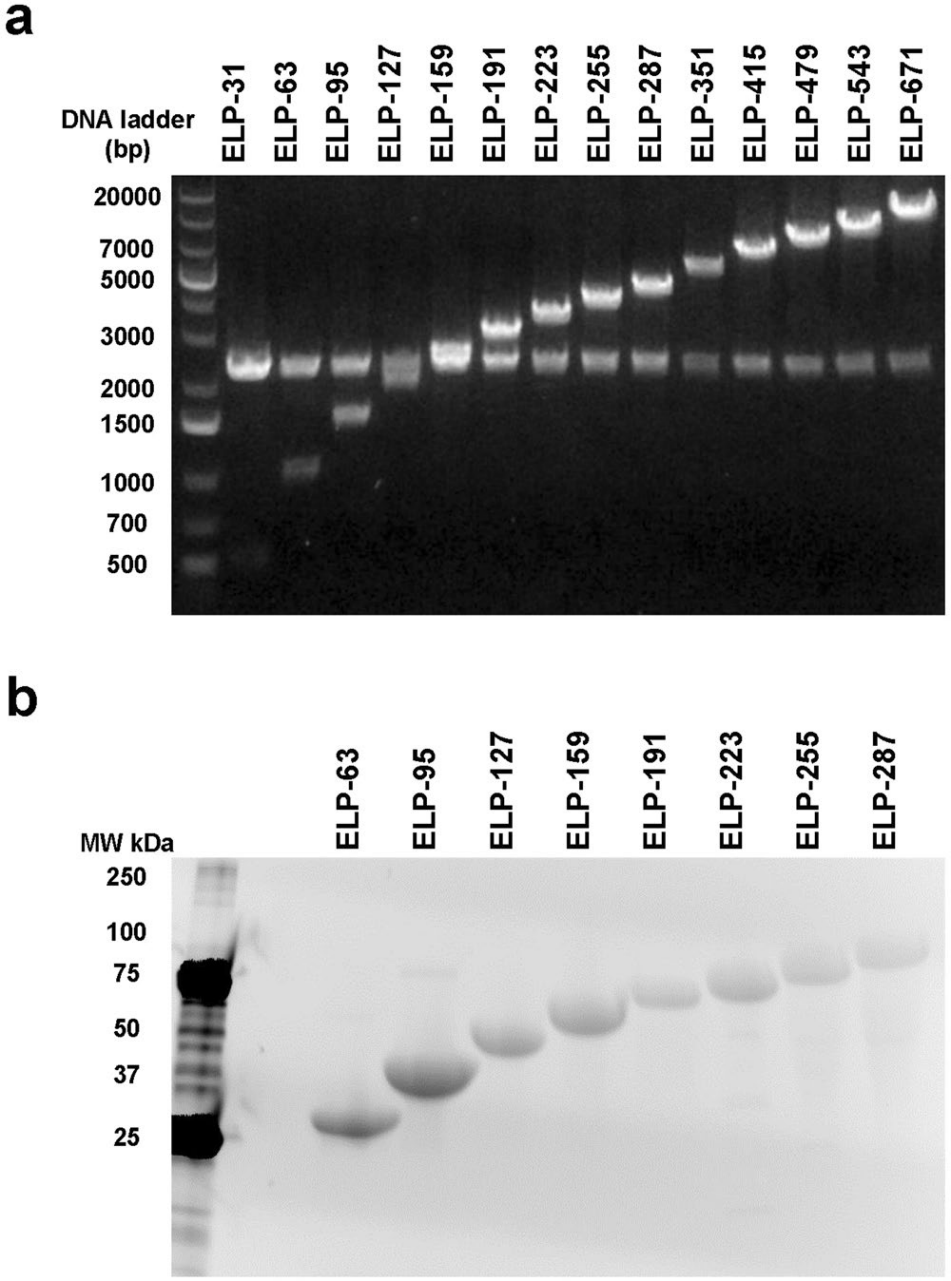
Assessment of ELP Expression Constructs and Protein Expression. (**a**) ELP coding DNA was digested and insert size evaluated on an agarose gel. The band at 2.5 kb is the vector backbone, and the band increasing in size is the ELP insert. (**b**) ELP protein purity was assessed by SDS-PAGE and visualized using fluorescence imaging of Mini-PROTEAN TGX Stain-Free gels.

We designed ELPs with varying coding sequence sizes ranging from 480 bp to 10,080 bp (Fig. 1a). These constructs encoded for proteins with a MW range from 13 to 256 kDa. A full list of constructs is shown in Table 1. The recombinant expression system we used was sufficient for successful purification of the proteins with sizes between 25 kDa and 110 kDa. The proteins were obtained at high purity, and each migrated at the expected molecular weight on an SDS-PAGE gel (Fig. 1b). The transition temperature of the smallest protein, ELP-31, was too high to achieve efficient thermal precipitation. ELP proteins above 110 kDa did not express with high efficiency under our standard culture conditions, likely due to their very long length and high concentration of the specific amino acids valine, proline, and glycine.

**Table 1:** ELP constructs, their coding sequence size, and predicted protein molecular weight.

Protein	Number of VPGxG repeats	Insert size (bp)	Number of amino acid residues	Predicted protein MW (kDa)
ELP-31	31	480	170	13.0977
ELP-63	63	960	330	25.2475
ELP-95	95	1440	490	37.3972
ELP-127	127	1920	650	49.5469
ELP-159	159	2400	810	61.696
ELP-191	191	2880	970	73.8463
ELP-223	223	3360	1130	85.996
ELP-255	255	3840	1290	98.1457
ELP-287	287	4320	1450	110.2955
ELP-351	351	5280	1770	122.4452
ELP-415	415	6240	2090	158.8943
ELP-479	479	7200	2410	183.1937
ELP-543	543	8160	2730	207.4932
ELP-671	671	10080	3370	256.092

### *In Vitro* Characterization of Purified ELP Proteins

**Figure 2:**
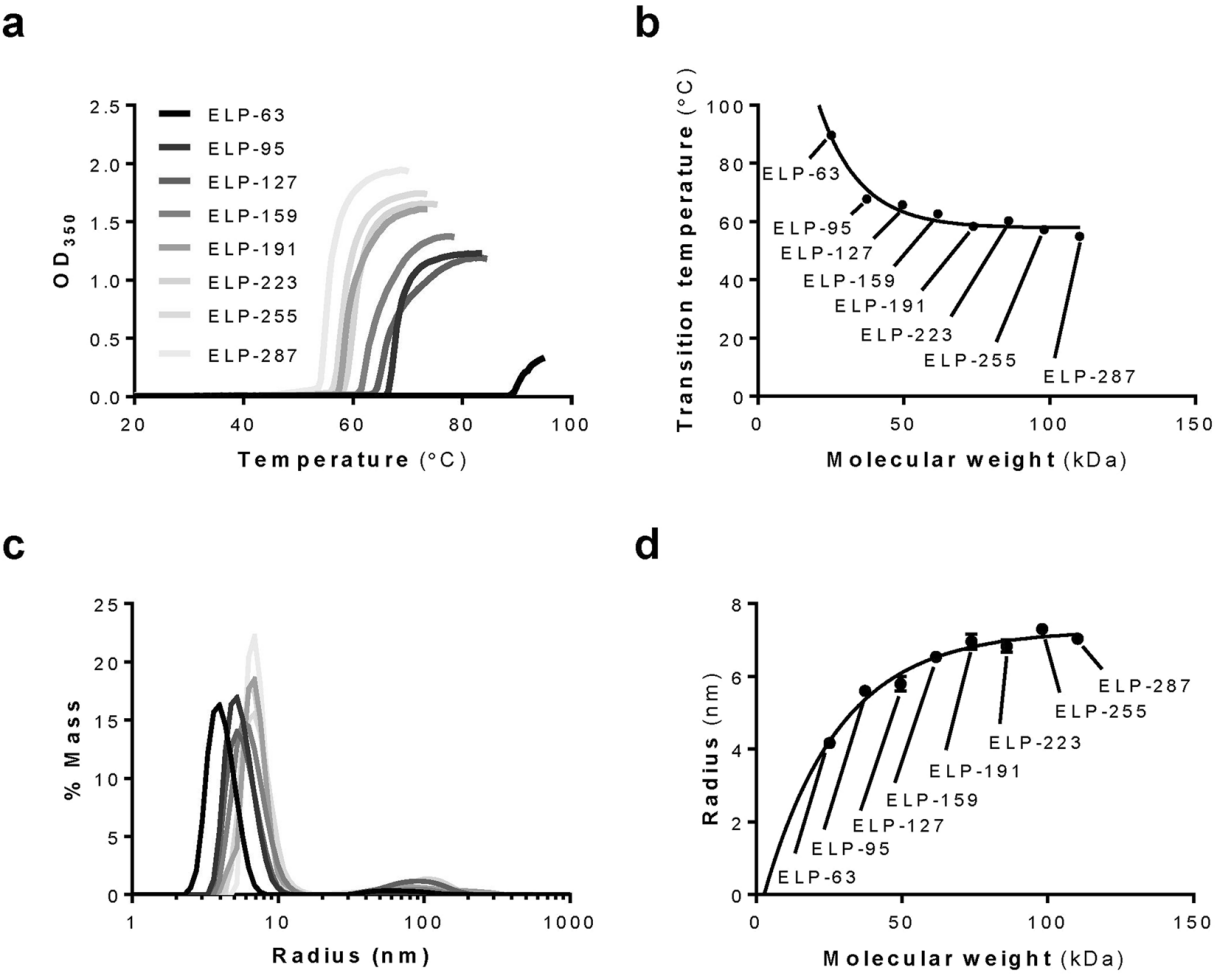
Determining the Transition Temperature and Hydrodynamic Radius of ELP Constructs. (**a**) Turbidity profiles (Abs 350 nm) of ELP proteins (10 µM in PBS, 0.22 µm filtered), obtained at a heating rate of 0.5 °C/min. (**b**) Transition temperature T_t_ as a function of ELP molecular weight (MW) fit by nonlinear regression using Prism (GraphPad) to a one-phase exponential decay function. T_t_ is determined as the peak of the first derivative of turbidity. (**c**) Radius, size distribution and estimated relative amount of mass in each peak of species obtained by dynamic light scattering. (**d**) Hydrodynamic radius R_h_ as a function of ELP MW fit by nonlinear regression using Prism (GraphPad) to a one-phase exponential function.

Following purification, proteins ranging from 25 kDa to 110 kDa were characterized *in vitro* to determine their transition temperature (T_t_) and hydrodynamic radius (R_h_) by turbidity assay and dynamic light scattering, respectively. With an increase in MW, the T_t_ of each protein decreased until it neared an asymptote at 54 °C for the 110 kDa ELP (Fig. 2a–c). Their radius increased with an increase in MW in the size range from 25 kDa to 110 kDa. Yet, similar to what was observed for T_t_, hydrodynamic radius reached an asymptote for the 98 kDa and larger proteins at 7 nm (Fig. 2d–f). Detailed T_t_ and hydrodynamic radius data are reported in Table 2.

**Table 2:** Parameters of ELP constructs obtained by turbidity and dynamic light scattering assays.

Protein	Predicted protein MW (kDa)	Transition temperature (°C)	Radius (nm)
ELP-63	25.2475	89.745	4.170 ± 0.056
ELP-95	37.3972	67.795	5.600 ± 0.030
ELP-127	49.5469	65.775	5.800 ± 0.200
ELP-159	61.696	62.745	6.530 ± 0.115
ELP-191	73.8463	58.370	6.967 ± 0.208
ELP-223	85.996	60.250	6.830 ± 0.169
ELP-255	98.1457	57.295	7.300 ± 0.100
ELP-287	110.2955	54.920	7.033 ± 0.058

### Assessment of ELP Stability *In Vitro*

**Figure 3:**
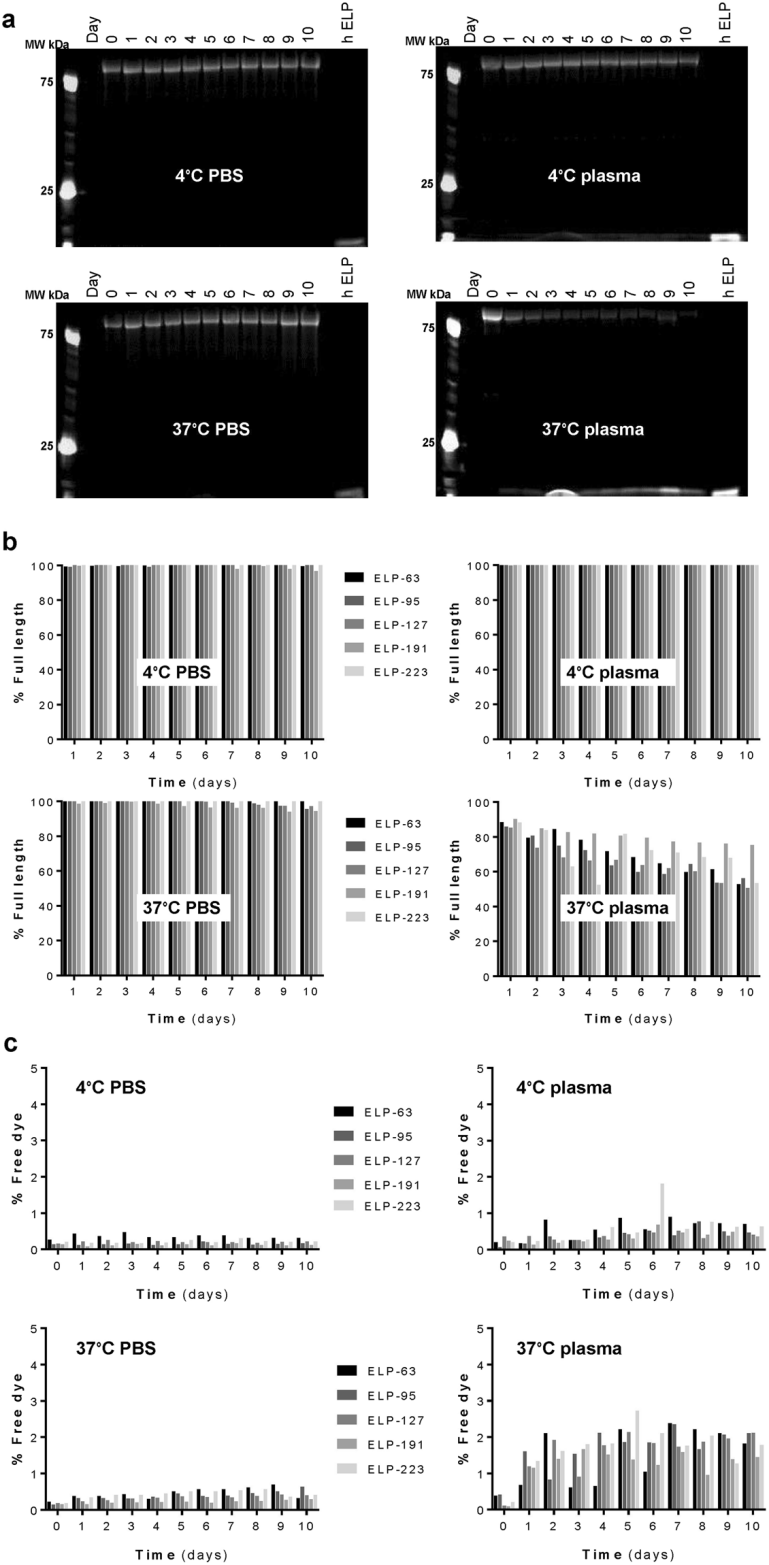
Stability of ELP Constructs. (**a**) Representative SDS-PAGE gel for ELP-223 (86 kDa) with hydrolyzed ELP (hELP) as a positive control, visualized by direct fluorescence imaging of the fluorescently-labeled ELP. (**b**) Polypeptide stability quantified from the SDS-PAGE analysis for all sizes of ELP proteins. (**c**) Fluorophore loss evaluated by direct fluorescence measurements.

ELP stability was assessed *in vitro* by determining the percent of fluorescently labeled full length polypeptide present after up to 10 days of incubation in either PBS or plasma at 4 or 37 °C, and by determining the percent of dye released from the polypeptide. Five ELP proteins were selected with a range of MW from 25 to 86 kDa. An example gel showing the results from the 86 kDa protein is shown in Fig. 3a. All polypeptides proved to be stable in PBS at both 4 and 37 °C, and in plasma at 4 °C, with only minimal degradation detected at very late time points (Fig. 3b). Degradation of the polypeptides was observed when incubated in plasma at 37 °C (Fig. 3b, lower right). About 80–90% of the proteins were still present as full-length protein on day 1, and each showed a slow degradation over the ten-day time course. On day 10 for ELP-63, ELP-95, ELP-127, ELP-191 and ELP-223, the percent of full length was 53, 56, 51, 75 and 53%, respectively. Dye release did not exceed 4% in conditions tested (Fig. 3c), even in the 37 °C plasma samples, indicating that even after significant degradation had occurred, the dye was still bound to a protein component.

### Plasma and Tissue Clearance Pharmacokinetics of ELP Proteins

**Figure 4:**
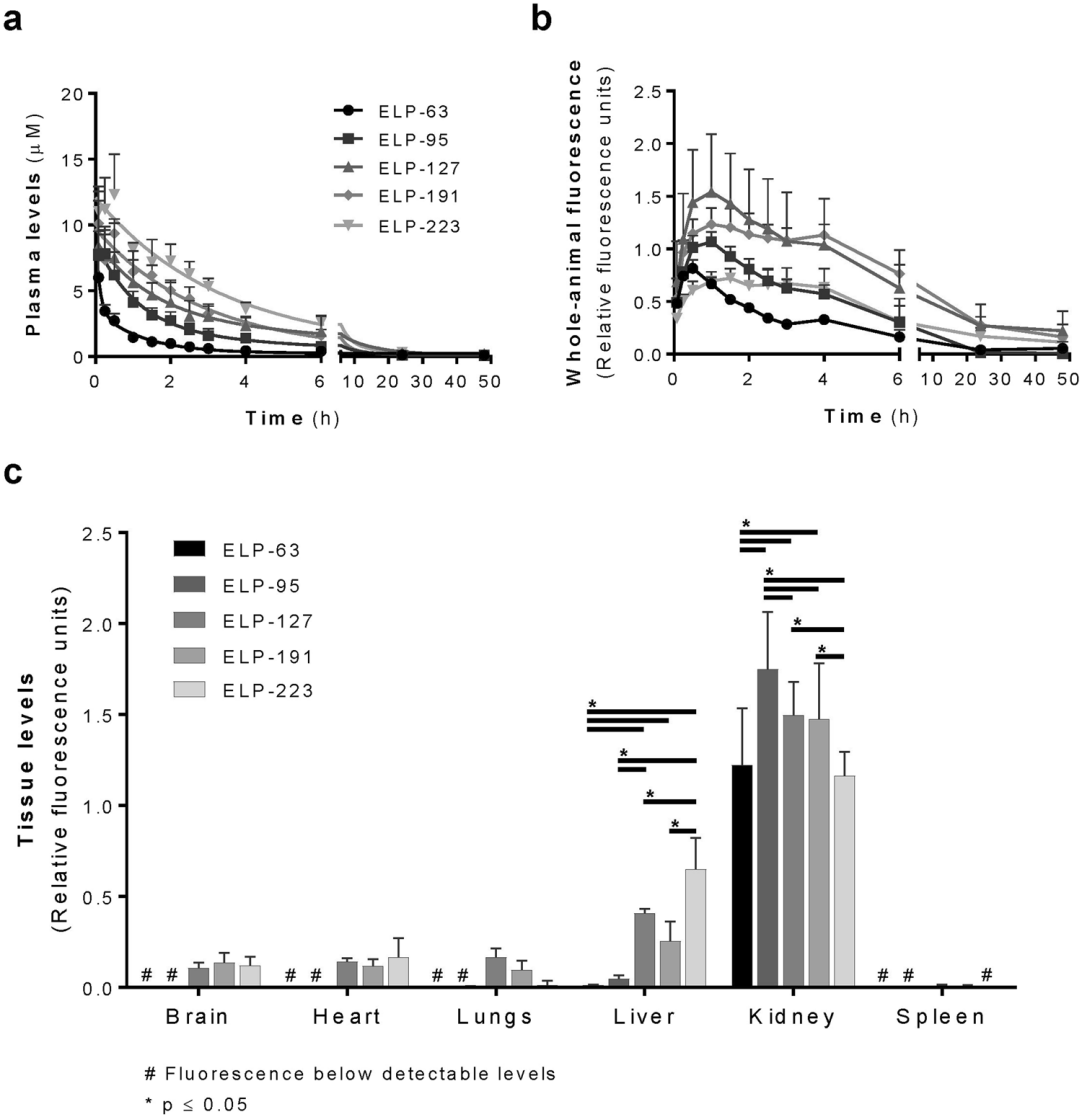
Plasma and Tissue Pharmacokinetics and Tissue Biodistribution of ELP Constructs. (**a**) Plasma levels of ELP proteins with fit lines representing a two compartment pharmacokinetic model fit. (**b**) Whole-animal clearance kinetics determined by non-invasive *in vivo* imaging of entire mice at each time point. (**c**) ELP tissue biodistribution 4 hours after administration. Values are mean ± SD, n = 4, except ELP-127 chronic study where n = 6. *Statistically significant difference between indicated groups as assessed by a two-way ANOVA with post hoc Tukey’s multiple comparison, p ≤ 0.05. ^#^Polypeptide levels below detectable levels.

A chronic biodistribution study was conducted in SKH1 Elite hairless female mice to determine the effects of MW on plasma pharmacokinetics and total tissue levels of ELP. Five ELP proteins were selected with a range of MW from 25 to 86 kDa. After bolus intravenous injection, plasma clearance was fit to a two-compartment pharmacokinetic model (Fig. 4a and Table 3). This study clearly demonstrated that an increase in MW resulted in slower plasma clearance. The terminal half-life of the smallest protein, ELP-63 (25 kDa), was 0.84 h, and was directly proportional to MW (Pearson’s correlation coefficient r = 0.9375, n = 5, p = 0.0186). The biggest protein, ELP-223 (86 kDa), had a terminal half-life of 16.99 h, a 20-fold increase. The distribution half-life was also directly proportional to MW (Pearson’s correlation coefficient r = 0.9929, n = 5, p = 0.0.0007). Interestingly, whole-animal fluorescence, depicting tissue levels of fluorescently labeled ELP, increased for the first 30 minutes after injection of the smallest ELP-63, then began to decrease as the protein cleared the body (Fig. 4b). Increasing MW lead to a shift of the tissue clearance curve to the right. ELP-63 peaked at 30 minutes and ELP-223 at 90 min. The ELP proteins with MW above 37 kDa cleared tissue more slowly and were still detectable in the body even 48 h after injection.

**Table 3:** Pharmacokinetics of ELP constructs in mice.

			ELP-63 (25 kDa)	ELP-95 (37 kDa)	ELP-127 (50 kDa)	ELP-191 (74 kDa)	ELP-223 (86 kDa)
Central Compartment Volume of Distribution	V_c_	*(L)*	0.004183	0.004996	0.00442	0.004603	0.00364
Plasma Clearance	Cl	$(\frac{L}{h})$	0.00801	0.00219	0.00105	0.00115	0.00079
Area Under Curve	AUC	$(\frac{\mu mol\times h}{L})$	4.76	19.83	37.15	38.72	55.92
Distribution Half-Life	t_1/2,dist_	*(h)*	0.07	0.77	1.07	1.97	2.27
Terminal Half-Life	t_1/2,term_	*(h)*	0.84	4.66	7.05	21.11	16.99

### Biodistribution of ELP Proteins

An acute biodistribution study was conducted to determine organ levels of ELP proteins with varying MW. Four hours after intravenous injection of fluorescently labeled ELP, mice were euthanized and major organs removed to quantify ELP tissue levels. As shown in Fig. 4c, all ELP proteins accumulated most strongly in the kidneys regardless of their MW. The smallest proteins, the 25 kDa ELP-63 and the 37 kDa ELP-95, had either very low or below detectable levels in the brain, heart, lungs, liver and spleen.

The liver is the only other organ where all five of ELP proteins were detected at noteworthy levels, and liver levels increased with increasing MW. The most remarkable finding was the effect of MW on deposition of ELP in the kidney. Renal deposition exhibited a non-linear relationship with MW (Pearson’s correlation coefficient r = −0.3079, n = 5, p = 0.6142, R^2^ = 0.09481), with the mid-sized proteins accumulating in the kidneys at the highest levels. ELP-63 levels, 1.22 relative fluorescence units (RFU), were significantly lower than ELP-95, 1.75 RFU, ELP-127, 1.49 RFU, and ELP-191 1.47 RFU. ELP-95 levels were additionally higher than ELP-127, ELP-191 and ELP-223 levels. ELP-127 levels were also significantly higher than ELP-223 levels, 1.16 RFU (Two-way ANOVA with post hoc Tukey’s multiple comparison, F(4, 90) = 8.74, p < 0.0001).

### Intrarenal Localization of ELP Proteins

**Figure 5:**
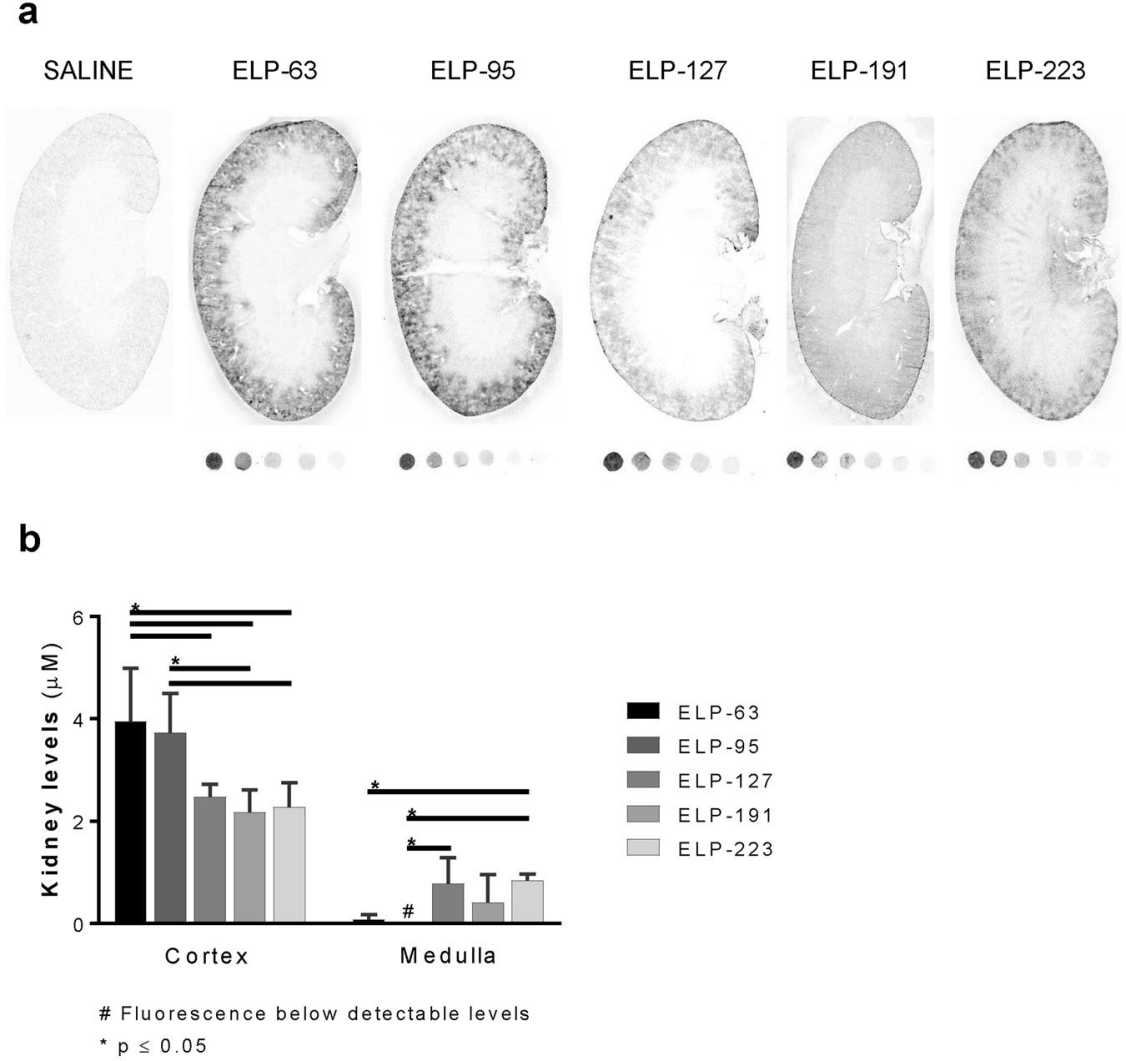
Quantitative Analysis of ELP Intrarenal Levels. Kidney ELP levels four hours after administration. (**a**) Fluorescence slide scans of the whole kidney section. (**b**) ELP levels in the cortex and medulla of the kidney sections as determined by quantitative fluorescence histology. Values are means ± SD, n = 4. *Statistically significant difference between indicated groups as assessed by a one-way ANOVA with post hoc Tukey’s multiple comparison, p ≤ 0.05. ^#^Polypeptide levels below detectable levels.

In addition to whole organ *ex vivo* imaging, we also performed quantitative fluorescence histology of kidney sections in order to accurately measure intra-renal concentrations and to determine the intra-renal distribution. Scans of kidney sections revealed that the smaller ELP-63 and ELP-95 localized exclusively in the renal cortex (Fig. 5a).

**Figure 6:**
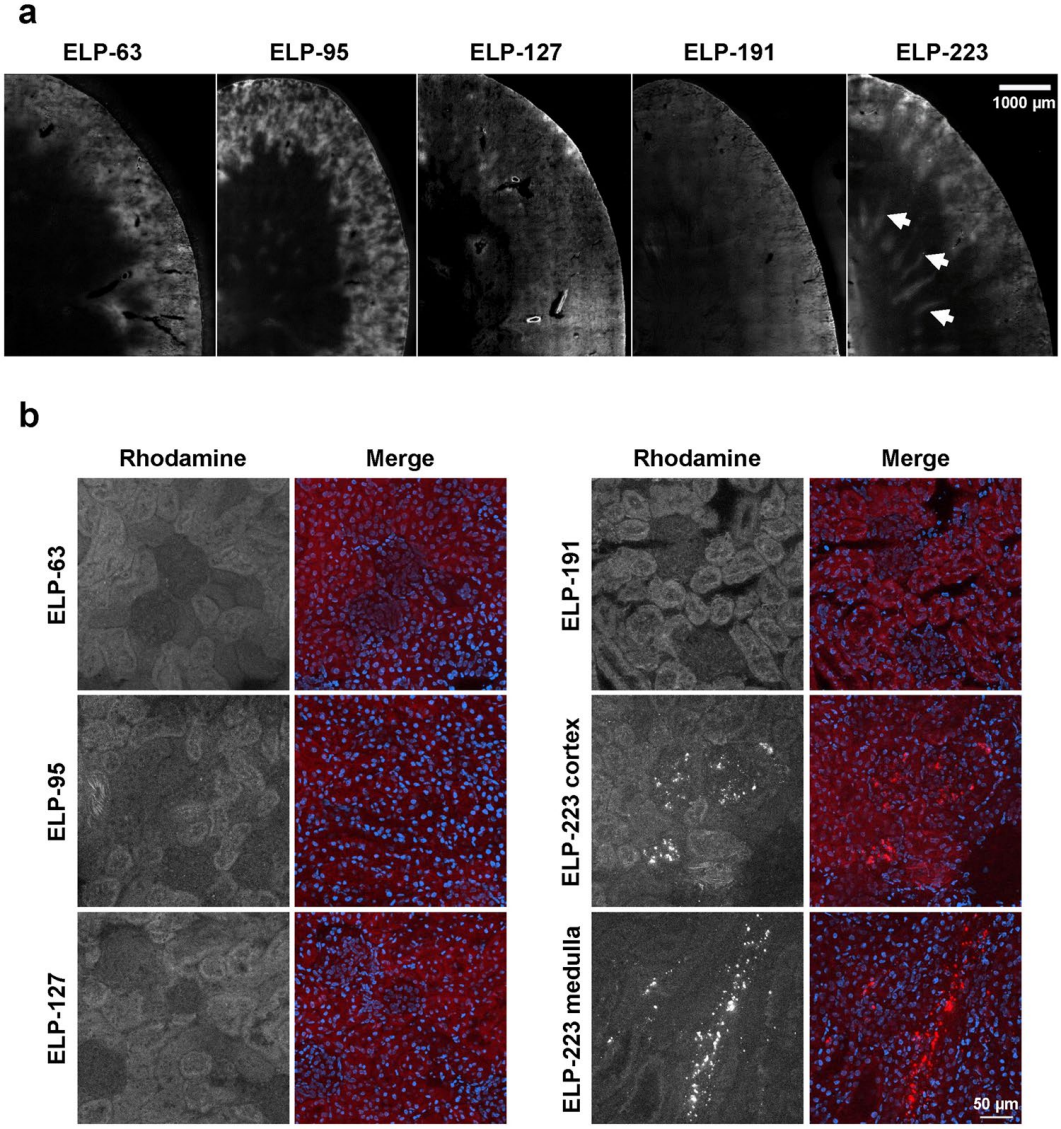
Intrarenal Distribution of ELP Constructs. (**a**) Intrarenal localization of ELPs in kidney sections obtained by confocal microscopy image stitching, scale bar 1000 µm. (**b**) Intrarenal localization of ELPs in kidney sections stained with Hoechst 33342 and imaged by confocal microscopy, scale bar 50 µm. Brightness levels were adjusted to optimize image quality and do not represent actual intensity.

With an increase in MW, the ELP proteins became more evenly distributed in the cortex and medulla. Quantitation of these data revealed that the cortical ELP concentration was highest for the smallest proteins, reaching an intra-cortical concentration of around 4 μM at the dose used, and significantly decreasing to around 2 μM for the largest proteins (one-way ANOVA with post hoc Tukey’s multiple comparison, F (4, 15) = 6.753, p = 0.0026; Pearson’s correlation coefficient r = −0.8938, n = 5, p = 0.0409). Concomitant with the decrease in cortical levels, the medullary ELP levels significantly increased as the polymer size increased (Fig. 5b), from around 0.07 µM for ELP-63 to around 0.84 µM for ELP-223 (one-way ANOVA with post hoc Tukey’s multiple comparison, F(4, 15) = 5.247, p = 0.0076; Pearson’s correlation coefficient r = 0.7325, n = 5, p = 0.1593). This was confirmed by confocal microscopy of unprocessed slides, shown in Fig. 6a. The smallest ELPs localized cortically and appeared to be mostly present in the renal tubules. As the size increased, the medullary levels increased, and the largest construct, ELP-223, was detectable in distinct medullary structures (Fig. 6a, arrows). Higher magnification imaging with nuclear co-staining revealed that in the cortex, all ELP proteins other than ELP-223 were mostly localized in the tubular epithelial cells, with lower levels in the glomeruli (Fig. 6b). The 86 kDa ELP-223, however, formed aggregates in the glomeruli, and high-resolution images revealed that the distinct medullary signal seen in the slide scanning data was actually protein aggregates in medullary structures (Fig. 6b, right panel middle and bottom).

## Discussion

Our study aimed to elucidate how molecular weight impacts pharmacokinetics, biodistribution and renal localization of the macromolecular therapeutic carrier, ELP. ELPs have been utilized in the past two decades for tumor targeted drug delivery^5,25,29^, for generation of slow-release subcutaneous drug depots^7,22,30^, for ocular drug delivery^10,31^, and to prevent placental drug transfer^8,32^ along with many other applications. Original work with ELPs was done in the cancer drug delivery field, and their tumor accumulation has been investigated extensively^5,15,25,29^. Yet ELP renal accumulation and filtration hasn’t been, to our knowledge, studied. Furthermore, given the growing field of ELP fusion therapeutics, a careful examination of how the polymer’s size affects its pharmacokinetics and biodistribution is warranted.

In one specific application, our lab has been focused on ELP utilization for delivery of growth factors for treatment of renovascular disease^9,28^, taking advantage of the fact that ELPs have an increased accumulation in kidneys^5,29^. Additionally, we are developing strategies that could improve renal accumulation such as ELP modification with a kidney targeting peptide (KTP)^33^. Similar strategies, such as low molecular weight proteins (LMWP) with MW below 30 kDa, have been utilized for targeting drugs to the kidneys^34^. Considering that our treatment strategy utilizes a fusion protein (ELP fused vascular endothelial growth factor or ELP-VEGF) with MW of 75 kDa^9,17,28^, a systematic study of the impact of MW on the biodistribution of these macromolecular carriers was needed.

We made a library of ELPs in a range of MW from 25 to 110 kDa and characterized their physical properties such as radius and transition temperature. The library was designed to have transition temperatures well above body temperature in order to allow *in vivo* systemic delivery without triggering the ELP phase transition and polypeptide aggregation. This allowed study of the effects of ELP MW directly without the confounding influence of ELP aggregation. T_t_ has been extensively studied by Meyer and Chilkoti^35^, who observed that the ELP T_t_ is inversely related to chain length. This finding was confirmed by our study as well. We also observed that increasing the ELP MW past 74 kDa does not decrease the T_t_ past 55 °C.

As our final goal was to study renal localization and filtration of these polypeptides, which is dependent not only on MW of these molecules but their size and shape as well, we measured their hydrodynamic radius by dynamic light scattering. These measurements provided the spherical equivalent radius of a hard sphere diffusing at the same rate as the particle of interest. Previous study by Fluegel *et al*. examined the hydrodynamic radius of several ELPs with varying MW, but they did not look at MW above 50 kDa^36^, a size range we were interested in due to its relevance in renal filtration. Our lower MW ELP proteins had radii consistent with data reported by Fluegel *et al*., however the most striking observation in our study was that the radius reached an asymptote where further increase in MW did not result in a further increase in radius. Bearing in mind that ELPs belong to a group of intrinsically disordered proteins, and are linear polymers with structure widely reported as β-spiral^13^ this observation was unexpected. Future studies will shed light on detailed biophysical characterization of this protein library.

In addition to issues of size and shape, with the respect to future plans to use these proteins for therapeutic delivery, we performed *in vitro* stability assays which showed that the rate of degradation is not MW dependent. These data confirmed that ELP as a macromolecular carrier is stable in various conditions, and the rate of degradation of these proteins in plasma at body temperature is slower than the rate of clearance previously reported^33^ and the clearance rate observed in our pharmacokinetic study. Therefore, even though degradation of these polypeptides was increased in biological conditions, degradation was slow and should have minimal effects on their *in vivo* fate.

Chronic biodistribution studies examined the impact of MW on pharmacokinetics of these polypeptides. As expected based on literature^26^, the terminal half-life increased with an increase in MW, with the largest 86 kDa ELP terminal half-life being 20-fold higher than the smallest 25 kDa ELP. A peak in whole-animal tissue levels was observed shortly after injection, and the time to peak levels was shifted to the right with an increase in MW, suggesting that larger proteins had a slower tissue uptake when compared to smaller proteins. These whole-animal fluorescence data complemented the plasma pharmacokinetics to suggest that bigger ELPs exchanged between plasma and tissues and cleared the body more slowly that smaller ELPs.

Biodistribution studies showed that all ELPs in our size library preferentially accumulated within the kidneys, consistent with previous *in vivo* drug delivery applications using other types of ELP fusion proteins^5^. Kidney levels were lower for the smallest and largest ELPs, while the medium sized ELPs had increased kidney levels. In addition, liver levels increased with an increase in MW. Careful analysis of kidney deposition revealed that kidney cortical level decreased and medullary levels increased with an increase in MW. Knowing from whole-animal levels that smaller proteins have faster tissue uptake and release, we can conclude that the same will happen in kidney tissue. Smaller proteins most likely are rapidly filtered, but then are reabsorbed in the proximal tubule, leading to the predominantly cortical signal. Larger proteins, on the other hand, may have a slower rate of filtration and/or a slower rate of tubular reabsorption, which would explain why there is an increase in medullary levels with an increase in MW. Future studies will seek to directly observe ELP renal filtration and intra-renal trafficking. A surprising finding was the largest ELP tested formed intra-renal aggregates that were about 2 µm in radius both within glomeruli and in medullary structures. Future histological staining is needed to determine the nature of this medullary signal. Slower filtration could account for aggregates in the glomeruli for ELP-223, while slower or no reabsorption could explain an increase in its medullary signal. However, it is unlikely that these aggregates pass the glomerular filtration barrier, so it is possible that larger ELPs are prone to aggregation *in situ*, especially in areas of high ELP concentration or high salt and urea concentration. It is important to note that the T_t_ of even this largest ELP was well above body temperature, so these aggregates are not likely to be the result of the canonical ELP phase transition – induced aggregation, unless they are in a renal structure with high salt concentration, which would decrease the T_t_ significantly. Though we have not observed any effect of our previously tested ELPs (around 60 kDa MW) on renal function even at very high doses^33^, these data may present a cautionary tale for using very large ELPs for systemic drug delivery, as aggregation in the kidney could reduce therapeutic potency.

Taken as a whole, these data suggest that lower molecular weight proteins are probably quickly filtered and reabsorbed within the kidney proximal tubule (where they may be degraded, but future work is needed to determine this) and likewise quickly cleared from the body. Therefore, they may be suitable for therapeutics designed to be short acting or to target disorders involving the proximal tubule. Medium molecular weight proteins (37–74 kDa) have longer plasma half-lives and higher total renal accumulation and might be better suited for use as systemically delivered biologics. Specifically, for renal delivery of therapeutic agents, the medium sized ELPs seem to be optimal owing to their longer terminal half-life, slower tissue clearance, and high renal deposition. Aggregation of the largest ELPs might be of concern because aggregates could slow targeting of the organ and may alter the interaction of ELP-fusion proteins with the cargo’s specific receptor, something that needs to be further examined in future studies. On the other hand, if aggregation does not interfere with target engagement, the slower filtration of these very large ELPs could provide for very long tissue residence times (and thus less frequent dosing) and more chances for the ELP cargo to bind to their targets, broadening the therapeutic window.

## Methods

### Synthesis of ELP Expression Constructs by Recursive Directional Ligation

We generated a new library of ELP constructs in which the ratio of amino acids at the X position in the V-P-G-X-G repeat is V:G:A in a 1:4:3 ratio. All ELP constructs are composed of pentapeptide repeats (VPGxG)_n_ denoted ELP-n, where n is the number of pentamer repeats. DNA encoding the ELP-31 sequence in a p-MA-RQ plasmid was custom synthesized (Life Technologies), and all subsequent constructs were synthesized by recursive directional ligation^19^. Each new ELP coding sequence was inserted into the *SfiI* site of a pET25b+ expression vector (provided by Drazen Raucher)^37,38^ encoding a short N-terminal sequence containing a cysteine residue and short C terminal sequence, resulting in a final coding sequence of MCGPG(VPGxG)nWPGSG, where n is 31 to 671 pentamer repeats. All constructs were confirmed by DNA sequencing (Eurofins Genomics).

### Purification of ELP Proteins

pET25b+ vectors encoding ELP proteins were transformed into *E. coli* BLR (DE3). All proteins were purified by inverse transition cycling, as described previously^17,39^. Briefly, 500 mL of *E. coli* BLR (DE3) bacterial cultures were grown in TB dry media for 18–20 hours in 2 L flasks. Cells were harvested by centrifugation, lysed by sonication, and nucleic acids were precipitated with polyethyleneimine and removed by centrifugation. NaCl was added to the soluble lysate to a concentration of 200 mg/mL, and the solution was heated at 47 °C until the ELP precipitated. The precipitated ELP was collected by centrifugation, re-dissolved in cold PBS, centrifuged at 4 °C to remove any un-dissolved precipitate, and this heat cycling process was repeated 2 times. ELP was once more precipitated and re-dissolved in a cold solution of 25% ethanol in PBS, centrifuged at 4 °C to remove any un-dissolved precipitate, precipitated again and resuspended in cold PBS. Purity was assessed by SDS-PAGE on a 4–20% Mini-PROTEAN TGX Stain-Free gel.

### Fluorescent Labeling of ELP Proteins

Each ELP protein was labeled on its N-terminal cysteine residue using a maleimide conjugate of rhodamine as described by Bidwell and Raucher^39^. Proteins were diluted to 200 µmol/L in 50 mM NaH_2_PO_4_ pH 7 buffer, and tris-(2-carboxyethyl)phosphine (TCEP) was added to a 10-fold molar excess. Tetramethylrhodamine-5-maleimide (Molecular Probes) was added to a 2-fold molar excess and the reaction was allowed to proceed overnight at 4 °C. Unreacted dye was removed by multiple washes with an Amicon 3,000 molecular weight cutoff spin filter (Merck Millipore). Labeling efficiency was assessed by UV-visible spectrophotometry (NanoDrop 2000, Thermo Fisher Scientific, Waltham, MA), a method modified from Bidwell and Raucher^39^. Removal of unreacted label was confirmed by trichloroacetic acid (TCA) precipitation of the labeled protein and assessing the free fluorophore levels in the supernatant spectrophotometrically.

### Determining the Transition Temperature of ELP Constructs

ELP samples in phosphate buffered saline were filtered through a Millex-GV hydrophilic Durapore (PVDF) filter with a pore size of 0.22 µm. 10 µM of filtered protein solution was heated at a constant rate of 0.5 °C/min in a temperature-controlled multicell holder in a UV-visible spectrophotometer (Cary 100), and the turbidity of the solution was measured as absorbance at 350 nm. The transition temperature (T_t_) was determined as the temperature at which a maximum was observed in a plot of the first derivative of the turbidity trace using GraphPad Prism version 7.00 for Windows.

### Determining the Hydrodynamic Radius of ELP Constructs

10 µM of filtered (0.22 µm) protein solution was evaluated by dynamic and static light scattering using DynaPro NanoStar (Wyatt Technology) with laser wavelength of 663.53 nm. Batch measurements were performed at a constant temperature of 20 °C, the signal acquisition period was set to 5 s, and an averaged result of 10 acquisitions was taken as a measurement. A total of 3 measurements was done. The refractive index increment dn/dc for protein was set to 0.185. Data were analyzed using Dynamics software (Wyatt Technology) using a Mw-R model of linear polymers and a static light scattering conformation model of random coil. Radius (nm) and %Mass were expressed as the mean value of the peak of the size distribution from the Regularization Graph using the Coils model in Dynamics.

### Determining the Stability of ELP Constructs

To determine the stability of polypeptides, 50 µM of each fluorescently labeled ELP was incubated in PBS or plasma at 4 or 37 °C for up to 10 days. Fluorophore loss from polypeptides was assessed by measuring fluorescence before and after precipitation of the proteins with 20% TCA. Fluorescence levels after TCA were corrected for dilution and compared to the pre-precipitation fluorescence to calculate the percentage of free dye at each time point. Polypeptide degradation was further assessed by SDS-PAGE on a Bolt 4–12% Bis-Tris Plus gels in reducing conditions for PBS samples and non-reducing conditions for plasma samples. Gels were visualized by direct fluorescence imaging using an IVIS Spectrum (PerkinElmer) and analyzed using Living Image Software. Fluorescence was measured as total radiant efficiency for both the total lane area including the ELP band and the lane area under the ELP band. The percentage of the sample that was degraded was determined by dividing the band intensity below the ELP band by the total band intensity. All calculations were corrected by the signal present at time 0 in order to account for any signal present as lower molecular weight species prior to the incubations. As a control, fluorescently labeled protein was hydrolyzed using a method modified from Zhong *et al*.^40^. 15 µM of fluorescently labeled protein was resuspended in 500 µl of 25% aqueous trifluoroacetic acid (TFA) solution. 10 µl of protein solution was placed in 1.5 ml polypropylene centrifuge tube, capped and sealed with a Teflon tape. Sample was microwave irradiated for 10 min, followed by vacuum centrifugation (Savant Speed Vac Concentrator) to remove the acid which was repeated until an adequate amount of the protein was hydrolyzed. Hydrolyzed protein was resuspended in H_2_O, and the sample was prepared for SDS-PAGE analysis.

### Pharmacokinetic studies

Animal studies were approved by the Animal Care and Use Committee of the University of Mississippi Medical Center and conducted according to the guidelines of the Guide for the Care and Use of Laboratory Animals^41^. For pharmacokinetic and biodistribution experiments, five ELPs ranging in MW from 25 to 86 kDa were used. SKH1-Elite hairless female mice (Charles River) were anesthetized with isoflurane (1–3%, to effect), administered carprofen (5 mg/kg subcutaneous), and injected with rhodamine-labeled polypeptides (1.5 µmol/kg) by intravenous injection into the femoral vein. Blood was sampled by tail prick intermittently for 48 hours, collected in Greiner Bio-One MiniCollect capillary blood collection tubes, and plasma was collected after centrifugation.

Plasma samples were analyzed for concentration of the polypeptides using quantitative fluorescence analysis. The fluorescence intensity of 2 µl of plasma was measured in a fluorescence plate reader on a NanoQuant Plate (Tecan) using an excitation wavelength of 535 nm and an emission wavelength 585 nm with Magellan software. Fluorescence of the plasma samples was compared to standard curves generated from known concentrations of the injected protein, which allows for comparison of multiple proteins regardless of the fluorescence labeling efficiency of each. A two-compartment model was fitted to the pooled data (mean concentration ± SD versus time; n = 4 except ELP-127 where n = 6) to develop a predictive mathematical model of the plasma concentration versus time.

Whole body fluorescence was measured at the same time as each blood sample by collecting dorsal view images of the live animal using an IVIS Spectrum. Images were collected using 535-nm excitation and 580-nm emission filters, autoexposure, and small binning. Using Living Image software, regions of interest were drawn over the entire animal, and mean radiant efficiency was measured to determine whole body fluorescence intensity. Standard curves of each injected protein were pipetted into a black 96-well plate, which was subsequently imaged with identical imaging parameters. Mean tissue fluorescence was fit to these standard curves to correct for any differences in labeling levels among polypeptides.

### Biodistribution and intrarenal localization of ELPs

For acute tissue biodistribution studies, SKH1-Elite hairless female mice were anesthetized with isoflurane (1–3%, to effect), administered carprofen (5 mg/kg subcutaneous), and given a single bolus dose of rhodamine-labeled polypeptides (1.5 µmol/kg) by intravenous injection into the femoral vein. Mice were allowed to rouse from anesthesia and move freely in the cage for four hours following injection. They were then re-anesthetized and euthanized while still under anesthesia, and their organs collected for whole organ fluorescence biodistribution analysis (n = 4 mice per agent). All major organs were imaged *ex vivo* using an IVIS Spectrum. Tissues were then embedded in freezing medium (Tissue-Plus O.C.T Compound) and flash frozen as described by McGowan^42^. Kidneys were cut into 14 µm sections with a cryostat. Sections were first scanned using a fluorescence slide scanner ScanArray Express (Packard BioScience) using excitation wavelengths 543 nm and emission wavelength 570 nm, scan resolution 50 µm, and full scan speed for quantitative scans; and scan resolution 5 µm and half scan speed for high resolution scans. For quantitative scans, the mean fluorescence intensity of tissue sections was analyzed with ImageJ software, and the measured fluorescence intensity was fit to a standard curve of each protein (made from known concentrations of the same labeling batch used for animal injections) prepared as described by McGowan^42^.

Sections were further analyzed by confocal microscopy. Slides were equilibrated to room temperature and either stained with Hoechst 33342 (5 µg/ml in PBS) or imaged without processing. Stained sections were covered by a coverslip, sealed and imaged immediately by laser scanning confocal microscopy (Nikon C2+) using 405- and 561-nm lasers for excitation of Hoechst 33342 and rhodamine-labeled protein, respectively. Unprocessed sections were imaged by confocal microscopy image stitching using 561-nm laser. Brightness levels were adjusted for image quality and don’t represent actual intensity.

### Statistical analysis

Organ biodistribution was assessed with a two-way ANOVA for factors of polypeptide treatment and organ type with post hoc Tukey’s multiple comparison. Kidney levels were assessed for differences between treatment groups with a one-way ANOVA with post hoc Tukey’s multiple comparison. Correlation was evaluated by Pearson’s correlation coefficient. All analyses were done using Prism (GraphPad), and a p value of <0.05 was considered statistically significant.

### Data availability

Experimental data are available from the authors upon request.
